# Correction: A durable monolithic polymer foam for efficient solar steam generation

**DOI:** 10.1039/c8sc90011f

**Published:** 2018-01-23

**Authors:** Qiaomei Chen, Zhiqiang Pei, Yanshuang Xu, Zhen Li, Yang Yang, Yen Wei, Yan Ji

**Affiliations:** a MOE Key Laboratory of Bioorganic Phosphorus Chemistry & Chemical Biology , Department of Chemistry , Tsinghua University , Beijing 100084 , China . Email: jiyan@mail.tsinghua.edu.cn; b Simpson Querrey Institute for BioNanotechnology , Northwestern University , Chicago , Illinois 60611 , USA

## Abstract

Correction for ‘A durable monolithic polymer foam for efficient solar steam generation’ by Qiaomei Chen *et al.*, *Chem. Sci.*, 2018, DOI: ; 10.1039/c7sc02967e.



## 

One of the funding numbers in the first sentence of the acknowledgements section was in error. The corrected version is shown below:

This research was supported by the National Science Foundation of China (no. 21674057 and 21274075).

The Royal Society of Chemistry apologises for these errors and any consequent inconvenience to authors and readers.

